# Magnetocaloric materials with ultra-small magnetic nanoparticles working at room temperature

**DOI:** 10.1038/s41598-019-53617-0

**Published:** 2019-11-26

**Authors:** M. R. Dudek, K. K. Dudek, W. Wolak, K. W. Wojciechowski, J. N. Grima

**Affiliations:** 1 Institute of Physics, University of Zielona Gora, ul. Szafrana 4a, 65-069 Zielona Gora, Poland; 2 Institute of Molecular Physics, Polish Academy of Sciences, M. Smoluchowskiego 17, 60-179 Poznan, Poland; 3 Metamaterials Unit, Faculty of Science, University of Malta, Msida, MSD 2080 Malta; 4 Department of Chemistry, Faculty of Science, University of Malta, Msida, MSD 2080 Malta

**Keywords:** Materials science, Magnetic properties and materials

## Abstract

Through the use of the Monte Carlo simulations utilising the mean-field approach, we show that a dense assembly of separated ultra-small magnetic nanoparticles embedded into a non-magnetic deformable matrix can be characterized by a large isothermal magnetic entropy change even upon applying a weak magnetic field with values much smaller than one Tesla. We also show that such entropy change may be very significant in the vicinity of the room temperature which effect normally requires an application of a strong external magnetic field. The deformable matrix chosen in this work as a host for magnetic nanoparticles adopts a thin film form with a large surface area to volume ratio. This in turn in combination with a strong magneto-volume coupling exhibited by this material allows us to show its suitability to be used in the case of a variety of applications utilising local cooling/heating such as future magnetic refrigerants.

## Introduction

In the last decade, it is possible to note a rapidly growing interest of the scientific community in the research related to applications involving the use of magnetic nanoparticles^1–18^. Among these studies, a particular attention has been devoted to the magnetocaloric property of the ultra-small nanoparticles (smaller than 50 nm) used for cooling purposes as discussed in the literature^2–6,9,11–13,15,17,19,20^.

Even though every magnetic material may exhibit a magnetocaloric effect (MCE), this effect is normally very weak at room temperature. Some exceptions include materials based on Gd, that has a critical temperature T_*c*_ = 294 K, several compounds based on manganites and rare earth metals^21^. It seems that a significant progress in this field originates from the discovery of a giant MCE in Gd_5_(Si_2_Ge_2_) by Pecharsky and Gschneidner^22^. Upon being inspired by this work, in the following years, many researchers made an attempt to identify other ferromagnetic materials that are capable of exhibiting a large MCE at room temperature. Such studies were focused primarily on adjusting the thermodynamic parameters of tested materials in a way so that the critical temperature T_*c*_ at which the ferromagnet-paramagnet phase transition takes place would be within the room temperature range. The example can be La_0.7_Ca_0.3−*x*_Ba_*x*_MnO_3_ compounds suggested for magnetic refrigeration technology near and above room temperature^23^ where the increase of T_*c*_ depends on the addition of the barium. Another example can be La_0.7_Ca_0.3−*x*_Sr_*x*_MnO_3_ magnetocaloric material^24^. The approach associated with the ferromagnet-paramagnet phase transition within the room temperature range ensures a large change in the value of the isothermal magnetic entropy Δ*S* at room temperature (MCE usually is described either by the isothermal entropy change Δ*S* or the adiabatic temperature change Δ*T*). Similar studies were also carried out for MCE materials with different types of magnetic phase transitions^25–30^.

Despite a possibility of observing a strong MCE effect at room temperature, for most materials it is necessary to apply a large magnetic field of a few Teslas to induce such effect which significantly limits its applicability. However, it does not have to be the case should one use materials based on systems composed of a sufficiently large number of magnetic nanoparticles as reported in experimental studies^2,4,20,31,32^. Furthermore, in the recent theoretical paper^33^, it was suggested that magnetic systems may exhibit a large magnetocaloric effect even without the presence of the external magnetic field. In this work, it was discussed that such effect can be achieved in the case of a deformable non-magnetic matrix having magnetic moments represented by Ising spins embedded into it. This means that the magnetocaloric effect can be controlled solely via the extent of an axial deformation of the matrix which process affects the interaction between magnetic inclusions. As suggested in^33^, in order to design such deformable matrix one can use mechanical metamaterials which are a class of materials that can exhibit unusual mechanical behaviour such as the negative Poisson’s ratio^34^ based on the way how they are designed. It should be also noted that the interest of the scientific community in these systems has grown rapidly in recent years due to various mechanical applications ranging from macroscale to nanoscale devices^35–39^. It is also worth to emphasise the fact that such materials offer the geometry-dependent strain-driven factor that can be utilised in an attempt of designing new types of magnetocaloric materials.

In the modern society, the control over the temperature of a specific object plays an important role in various aspects of our lives. Because of that, scientists devoted a lot of attention to studies related to the possibility of inducing the magnetocaloric effect in a specific small region where it could be used for local cooling with some examples of this approach being NEMS and MEMS devices^2^ and various biomedical applications^1^. In view of this, in this work, we consider a particular example of the thin magneto-auxetic film which we investigate from the point of view of its potential to induce the magnetocaloric effect. We also analyse its capability to exhibit this effect without the presence of a strong external magnetic field in order to assess its suitability to be used in the case of applications requiring local magnetic cooling.

## Model

**Figure 1:**
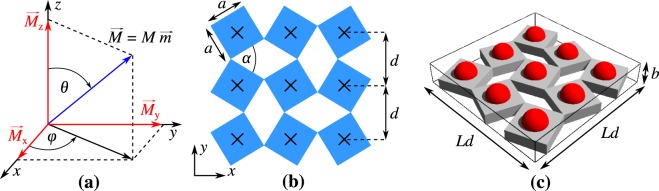
Panels show (a) a graphical representation of an example of the magnetic moment *M* associated with each of the nanoparticles within the system, (**b**) a projection of the surface of the non-magnetic deformable matrix composed of *L* × *L* = 3 × 3 structural units with the linear dimension defined as *a* where crosses indicate positions of embedded nanoparticles and (**c**) projection of the 3D layer of the auxetic material having a thickness denoted as *b* where red spheres represent magnetic nanoparticles.

In order to analyse the possibility of inducing a strong MCE in a magnetic nanoparticle system, we consider a model that was inspired by the recent work by the authors^33^. More specifically, we consider a thin deformable matrix consisting of non-magnetic rotating square-like units with magnetic inclusions located at the centre of each of them. In terms of the geometry of the non-magnetic matrix, the angle of aperture *α* between the structural units of the matrix determines a degree of its porosity and it may change upon stretching or compressing the system. Furthermore, magnetic inclusions considered in this work are assumed to be ultra-small single-domain magnetic nanoparticles having a uniaxial magnetic anisotropy where the way how they were incorporated into the non-magnetic matrix is shown schematically in Fig. 1. In all of the considered cases, magnetic inclusions were assumed to be Fe_3_O_4_ nanoparticles but it should be noted that the model used to generate the results, which is described in more detail in the following part of this work, is very general and it may also be used with other types of nanoparticles. Furthermore, it is assumed that each nanoparticle acts as a single superspin interacting with other superspins through dipolar interactions. Here it should be emphasised that the concept of the superspin representing a magnetic moment of single-domain magnetic nanoparticles is well-known in the literature and is usually used when describing superparamagnetism^40^. It should also be noted that in the case of dense arrangements of magnetic nanoparticles, as is the case in this work, magnetic nanoparticles can show a collective behaviour that is typical for spin glasses or superferromagnetic systems^40–45^. Furthermore, it should be emphasised that in this work, for the sake of simplicity, easy magnetization axes for all nanoparticles within the system are assumed to be oriented along the *z* axis.

In the considered model (see Fig. 1), the non-magnetic matrix is represented by a set of perfectly rigid rotating square-like units connected at their vertices by means of hinges that allow the system to deform. As shown in Fig. 1(b,c), each of the square-like units has a linear dimension of *a* and the out-of-plane thickness of the investigated material is denoted as *b*. Furthermore, as indicated in Fig. 1(a), each nanoparticle is represented by a vector with three components (*M*_*x*_, *M*_*y*_, *M*_*z*_) = (*Mm*_*x*_, *Mm*_*y*_, *Mm*_*z*_) where *M* is the magnetic moment of a nanoparticle and *m*_*x*_ = sin*θ*cos*ϕ*, *m*_*y*_ = sin*θ*sin*ϕ*, *m*_*z*_ = cos*θ*. The remaining symbols defined in Fig. 1, i.e. *d*, *a*, *θ*, *ϕ* and *α*, denote the distance between neighbouring nanoparticles, side length of a structural unit, polar angle defining the orientation of the magnetic moment in the *xy* plane, azimuthal angle describing the orientation of the magnetic moment with respect to the *z* axis and the angle of aperture between the neighbouring structural units of the non-magnetic matrix respectively. In view of this, the volume of a fragment of the analysed system containing *L* × *L* nanoparticles can be described as follows *V*_*s*_ = *b*(*Ld*)^2^.

There are several examples^14,37,39,46–48^ of metamaterials that may be considered as candidates to be used as a non-magnetic matrix similar to the one in Fig. 1 that can operate at room temperature. Moreover, some systems which could very well mimic the behaviour of the considered model having a fixed value of *α* were recently reported in experimental studies incorporating magnetic inclusions in the form of Gd_2_O_3_^5^ and Fe_3_O_4_^49^ nanoparticles that were embedded in silica matrix. Various magnetocaloric materials in a form of nanoscale thin films as well as engineering techniques used to prepare them can be found in the review article by Miller, Baley and Kirby^50^. Other examples of materials that are expected to have a potential to lead to a similar behaviour to the one discussed in this work can be Co/Au nanoparticle systems^17^, UV-cured magnetic polymer nanocomposities^51^ and magnetic nanoparticle superlattices^52^.

In the case of this work, the evolution of the nanoparticle-based system is analysed through the mean-field approximation (mfa) as in papers^53,54^ however in our case, the mean-field parameter is space-dependent instead of being uniform. An example of the quantum version of the mfa approach can also be found in^55^. Furthermore, it should be noted that in this work, the mfa Hamiltonian is applied to the system composed of *N* (*N* = *L*^2^) magnetic nanoparticles arranged on a square lattice (see Fig. 1) and is defined as follows:

1 $${H}_{{\rm{m}}{fa}}^{(i)}=-{K}_{a}V{\cos }^{2}{\theta }_{i}-BM\,\cos \,{\theta }_{i}-\sum _{j}\,{K}_{ij}{\overrightarrow{m}}_{i}\cdot  < {\overrightarrow{m}}_{j} > ,$$

where each magnetic nanoparticle at lattice site *i* is represented by a superspin having a magnetic moment ${\overrightarrow{m}}_{i}$ (${\overrightarrow{M}}_{i}=M{\overrightarrow{m}}_{i}$). We assume that *M* does not depend on temperature. The other of the used variables, i.e. *K*_*a*_ and *V*, are a uniaxial magnetic anisotropy energy constant and the volume respectively with both of these variables assuming a constant value for all nanoparticles. Furthermore, symbol *B* denotes an external magnetic field and *K*_*ij*_ represents magnetic interaction between nanoparticles located at the lattice site *i* and its nearest or the next nearest neighbour site *j*. The value of *K*_*ij*_ is chosen to be the mean dipolar energy *μ*_0_*M*^2^/(4*πd*_*ij*_^3^) between two magnetic nanoparticles with the center-to-center separation distance being equal to ${d}_{ij}=\sqrt{2}a\,\sin (\alpha /2+\pi /4.0)$ where *a* = 2*R*_*g*_ + *a*_0_ and *a*_0_ is the spacing between the neighbouring nanoparticles in a densely packed system (*α* = 0° or 180°). Symbol $\langle {\overrightarrow{m}}_{j}\rangle =\langle {\overrightarrow{M}}_{j}\rangle /M$ represents a mean-field magnetic moment at site *j* divided by *M*. It should also be noted that the magnetic field $\overrightarrow{B}$ is oriented towards the *z* axis similarly to easy-axes of all nanoparticles.

To calculate $\langle {\overrightarrow{m}}_{j}\rangle$ that appears in Eq. (1), we use the space-dependent mean-field Monte Carlo method which was initially proposed by Dudek *et al*.^56^ for one-body Hamiltonians. According to this method, one can assume that a given number *L*_*s*_ of independent copies of the system consisting of *N* magnetic moments ${\{{\overrightarrow{M}}_{i}\}}_{i=1}^{N}$ with the interaction Hamiltonian as in Eq. (1) is defined on the same lattice. Furthermore, each magnetic moment ${\overrightarrow{M}}_{i}$ can interact only with the mean fields ${K}_{ij}\langle {\overrightarrow{m}}_{j}\rangle$ at the neighboring lattice sites *j* which are the same for each copy and

2 $$\langle {\overrightarrow{m}}_{j}\rangle =\frac{1}{{L}_{s}}\mathop{\sum }\limits_{k=1}^{{L}_{s}}\,{({\overrightarrow{m}}_{j})}_{k},$$

where *k* denotes consecutive copies of the system. The evolution of the system towards thermodynamic equilibrium is based on the standard procedure incorporating the use of the Metropolis Monte Carlo algorithm and is described in the Methods section.

To determine the extent of the MCE for the considered system, it is necessary to calculate the change in its entropy. Fortunately, in the case of the mfa, correlations between neighbouring magnetic moments can be neglected and the Shannon entropy can be used. As a result, for each nanoparticle at site *i*, the magnetic entropy reads as follows:

3 $${S}_{i}/{k}_{B}={\int }_{0}^{2\pi }\,d\phi {\int }_{0}^{\pi }\,d\theta \,\sin (\theta ){p}_{i}(\theta ,\phi )\log \,{p}_{i}(\theta ,\phi ),$$

where *k*_*B*_ represents the Boltzmann constant. Furthermore, the factor sin(*θ*) in Eq. (3) is the Jacobian of the transformation from the Cartesian to the spherical coordinate system and *p*_*i*_(*θ*, *ϕ*) is the probability density describing the rotation of the magnetic moment ${\overrightarrow{M}}_{i}$ by an angle *θ* relative to the *z*-axis and angle *ϕ* with respect to the *x*-axis (see Fig. 1). Here, the expression describing *p*_*i*_(*θ*, *ϕ*) may be written down in the following manner:

4 $${p}_{i}(\theta ,\phi )=\frac{{{\rm{e}}}^{-\beta {H}_{{\rm{m}}{fa}}^{(i)}(\theta ,\phi )}}{{\int }_{0}^{2\pi }\,d\phi {\int }_{0}^{\pi }\,d\theta \,\sin (\theta ){{\rm{e}}}^{-\beta {H}_{{\rm{m}}{fa}}^{(i)}(\theta ,\phi )}},$$

where *β* = 1/*k*_*B*_*T*.

It should be emphasised that in the case of this work, the difficulty corresponding to the calculation of integrals in Eqs. (3) and (4) is overcome by choosing the Monte Carlo integration scheme (see Methods section). Furthermore, the total magnetic entropy per nanoparticle is defined as follows:

5 $$S/N{k}_{B}=(1/N{k}_{B})\mathop{\sum }\limits_{i=1}^{N}\,{S}_{i}.$$

Before analysing the extent of the change in the magnetic entropy of the investigated system, which process would allow to assess its suitability to be used in the case of magnetocaloric applications, it is important to first determine the maximum value of this quantity that can be assumed by the considered system. This stems from the fact that in the further part of this work, i.e. in the Results and Discussion section, the observed change in the entropy will be analysed through the comparison with such maximum value.

In the case of the considered system, the maximum value of the total magnetic entropy per nanoparticle (see Eq. (5)) is considerably large and can be estimated to be equal to *S*_*max*_ = *k*_*B*_log(4*π*) ≈ 2.5310 *k*_*B*_. This result can also be expressed in terms of the molar entropy as *S*_*max*_^(*u*)^ = *R*_*u*_log(4*π*) ≈ 21 J mol^−1^ K^−1^ with *R*_*u*_ being the universal gas constant (*R*_*u*_ = 8.3144621 J/mol K). It is also worth to note that some of the scientists focusing on studies related to the possibility of inducing the magnetocaloric effect quantify the extent of the change in entropy in terms of another unit, i.e. J kg^−1^ K^−1^. It should also be noted that in order to express the value of *S*_*max*_ in terms of this unit it is first necessary to define a particular representation of the general model shown in Fig. 1. To do such estimation, we assume that our system can be represented by a thin layer of silica having the out-of-plane thickness of *b* = 20 nm (see Fig. 1(c)) where magnetic nanoparticles inserted into such layer are Fe_3_O_4_ nanoparticles with a radius *R*_*g*_ = 3 nm. For such model, in the case of the particular configuration assumed by the system corresponding to the angle of aperture *α* = 0° (the separation distance between nanoparticles *d* is at its minimum), Fe_3_O_4_ nanoparticles constitute 0.13 mole fraction of the entire system. Furthermore, upon taking all of this information into consideration, it can be calculated that the maximum magnetic entropy of the considered system *S*_*max*_^*m*^ = *R*log(4*π*) ≈ 0.44 J kg^−1^ K^−1^ where *R* = *R*_*u*_/*M* and *M* stands for the molar mass associated with all constituents of the considered system. This number originates from a large molar mass of magnetic nanoparticles which is approximately 353.82 kg/mol and it represents about 1528 formula units per nanoparticle. For the sake of a comparison, it can be mentioned that for the same system corresponding to a very different geometric configuration where *α* = 90°, the maximum entropy increases up to *S*_*max*_^*m*^ ≈ 0.89 J kg^−1^ K^−1^. This means that the maximum entropy change can be estimated to be equal to Δ*S*_*max*_ = 0.45 J kg^−1^ K^−1^. At this point, it is important to emphasise the fact that these values are consistent in terms of the order of magnitude with experimental studies with some examples being the PNMA polymer film acting as a non-magnetic host for magnetic Fe_3_O_4_ nanoparticles which was suggested for local cooling/heating of NEMS and MEMS devices^2^ and ferrite nanoparticles used for similar purposes^3^.

At this point, it should be mentioned, that the model investigated in this work is not a purely hypothetical theoretical concept and that similar materials can be prepared in reality and tested through the use of the experiment. More specifically, as already discussed in more detail in this section, the model considered in this work consists of a mechanical lattice resembling the rotating squares system with structural units having magnetic nanoparticles embedded on them. Such mechanical lattice is used to change the distance between interacting magnetic nanoparticles. Thus, for magnetic interactions between nanoparticles not to be negligible, the structural elements of the lattice itself have to be appropriately small. This, in turn, may prove to be challenging from the practical point of view. However, in the literature, there are already known examples of very similar deformable lattices having their structural elements constructed in a way so that their dimensions are at the nano-scale. There are even examples of a nano-scale matrix in a form of the isotropic rotating squares system^48^ that exhibit the negative Poisson’s ratio equal to −1 irrespective of the stage of the mechanical deformation. This, in turn, describes the same deformation mechanism as the model discussed in this study. In the aforementioned work by Suzuki *et al*.^48^, the lattice was constructed through the use of mutants of the C_4_-symmetric protein RhuA that were intertwined together to form a crystalline array. Furthermore, the elasticity of the lattice allowing for the isotropic auxetic deformation was attributed to hinges utilising single-disulfide interactions. In view of this, the only challenge remaining to potentially construct the material resembling the theoretical model described in this work corresponds to the inclusion of magnetic nanoparticles on the lattice similar to the one proposed by Suzuki *et al*. It seems that this could be achieved in a number of ways with the most promising approach being a synthesis of nanoparticles directly on the surface of structural units of the lattice.

In addition to the fact that the considered model can be constructed in real life, which as already discussed stems amongst others from the fact that nonmagnetic matrices similar to the one discussed in this work were already reported in experimental studies, it is also worth to consider the effect that the presence of some of the factors appearing in real life while conducting experiments could have on magnetocaloric properties of the system. As the matter of fact, one of the most important of such factors is the demagnetization field. In one of the recent studies, Normile *et al*.^43^ indicated the need to take into account the effect of demagnetization in dense magnetic nanoparticle systems due to a sample shape. To this aim, they considered a densely packed powder of spherical *γ*-Fe_2_O_3_ magnetic nanoparticles in a form of a thin film with the thickness of around 2 mm and 20 *μ*m. The measured in-plane demagnetizing field factors were estimated to be even as large as 0.223 and 0.168 for the thick and thin nanoparticle assembly respectively. However, it should be also noted that the impact of the demagnetizing field on properties of the system can be decreased upon applying the external magnetic field along the magnetic easy axis direction^18,50^. A general discussion on dipolar interaction and the sample demagnetizing factors can be found in paper^57^. In the case of our model, the assembly of magnetic nanoparticles corresponds to nanoparticles that are not touching each other and magnetic easy direction is oriented in the orthogonal direction to the plane formed by the considered thin film which as mentioned above leads to a significant decrease of the effect that the demagnetization field has on properties of the system. In addition to that, the packing fraction Φ of the nanoparticles has a range from Φ = 0.067(*α* = 90°) to Φ = 0.13(*α* = 0°) depending on the aperture angle. It is also important to remember that the film shape does not change with the value of *α*.

## Results and Discussion

**Figure 2:**
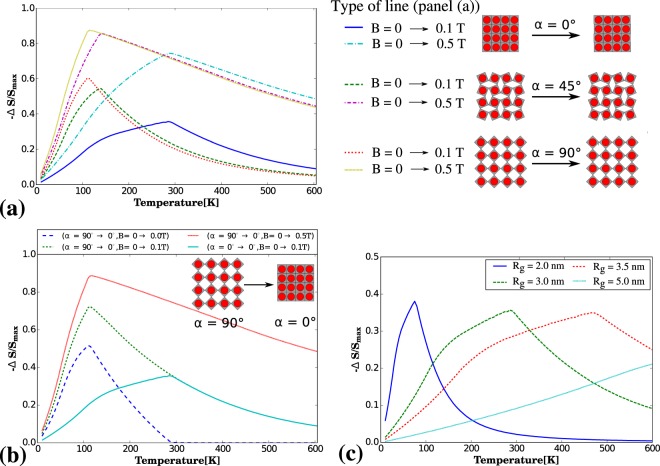
Graphs show isothermal change in magnetic entropy Δ*S* vs. temperature. In particular, the respective panels show (**a**) the variation in the extent of MCE for three systems of nanoparticles embedded on a non-magnetic matrix characterised by different values of *α*, i.e. *α* = 90°, 45° and 0°, that are subjected to a change in the external magnetic field of 0.1T and 0.5T, (**b**) the variation in the change of the entropy for systems subjected to the mechanical deformation corresponding to the change in *α* with the simultaneous application of the external magnetic field and (**c**) variation in the change of the entropy for systems having particles of different sizes embedded into a non-magnetic matrix. Parameters defined in the Model section that were used to generate the above results were set to be the following: *K*_*ij*_(*α* = 90°)/*k*_*B*_ = 35.11 K, *K*_*ij*_(*α* = 45°)/*k*_*B*_ = 44.53 K, *K*_*ij*_(*α* = 0°)/*k*_*B*_ = 99.32 K, *R*_*g*_ = 3 nm, *a*_0_ = 0.5 nm.

In this work, we analyse the capability of the considered system to induce a strong magnetocaloric effect through the use of the model described in the Model section. In particular, we investigate the effect that different factors such as the change in the geometric configuration assumed by the considered system and the variation in the external magnetic field has on the extent of the MCE which in this case corresponds to the variation in the isothermal magnetic entropy Δ*S* (see Fig. 2). As shown in Fig. 2(a), in all of the considered cases, the change in the external magnetic field results in a change in the entropy of the system as should be expected since it is a characteristic feature of all magnetic materials. However, it is interesting to note that for the same change in the external magnetic field (e.g. from 0 T to 0.1 T) the extent of the change in the entropy is different depending on the configuration assumed by the system. This stems from the fact that for different values of *α* the separation distance between interacting nanoparticles *d* changes as it was discussed in the Model section. Furthermore, upon comparing results corresponding to systems characterised by a different value of *α*, one can note that the maximum of −Δ*S*/*S*_*max*_ tends to shift towards low temperatures as the separation distance between nanoparticles increases. It should also be emphasised that based on the results presented in Fig. 2(a) it can be concluded that even small values of the magnetic field are sufficient for a very large change in the magnetic entropy, which is also the case in the vicinity of the room temperature.

In addition to the variation in the external magnetic field investigated for systems corresponding to specific values of *α*, it is very interesting to analyse the effect that the mechanical deformation corresponding to a change in the separation distance *d* has on the extent of the MCE. Similar concept was recently studied by Elouafi *et al*.^13^ where the effect of the change in the interparticle distance on the value of −Δ*S* was discussed. In this work, as shown in Fig. 2(b), the change in the entropy of the considered system can be induced solely by means of the mechanical deformation where in this case such deformation corresponds to the transition of the system from the configuration characterised by *α* = 90° (*d* is at its maximum) to the configuration where *α* = 0° (*d* assumes the minimum value). It is important to emphasise the fact that such effect can be induced even without the presence of the external magnetic field. However, the application of a change in the external magnetic field can further enhance the extent of −Δ*S*.

Another interesting aspect related to the considered model corresponds to the effect that a size of magnetic nanoparticles has on the extent of the MCE. It is particularly interesting to analyse this effect in the vicinity of the room temperature. As shown in Fig. 2(c), it can be noted that depending on the size of nanoparticles embedded into a nonmagnetic matrix, −Δ*S*/*S*_*max*_ changes in a different manner with temperature. Furthermore, one can realise that in the vicinity of the room temperature, there is a specific size of magnetic nanoparticles, i.e. *R*_*g*_ = 3 nm, in which case the maximum value of −Δ*S*/*S*_*max*_ can be observed. This means that should one consider the use of smaller or larger particles then the extent of the change in entropy would be smaller at such temperature. These results are consistent with a number of publications, e.g.^6,9,10^ which show that reducing the size of nanoparticles leads to a shift of the paramagnetic to ferromagnetic phase transition towards lower temperatures and, as a consequence, the maximum of Δ*S* is also moved towards lower temperatures. It should be also emphasised that the reduction of the dimensionality of the bulk magnetocaloric material to a nanoscale film form can significantly change the magnetic properties of these materials and, consequently, their magnetocaloric properties. The example can be the recent paper by Giri *et al*.^58^ where the strain induced magnetocaloric effect in La_0.67_Sr_0.33_MnO_3_ thin film is discussed. Another example is the effect of the reduction in the dimensionality of the bulk polycrystalline form of La_0.7_Ca_0.3_MnO_3_ to a thin film and nanocrystalline form reported by Lampen *et al*.^9^. Such change in the dimensionality was shown to lead to the change in the type of a phase transition which a given material can undergo. Lampen *et al*. managed to show that the first order phase transition observed in bulk La_0.48_Ca_0.52_MnO_3_ can be weakened in the case of a thin film with the nanocrystallines and it can be converted into the second order phase transition. In turn, in paper^59^, it was shown that antiferromagnetic bulk La_0.48_Ca_0.52_MnO_3_ compound can be converted into ferromagnetic material by reducing the particle size in nanometer scale. In this work, an increase in the magnetic entropy change following the reduction of the nanoparticle size was also observed.

It should be noted that in none of the considered cases the entropy change −Δ*S* did not reach the value of −Δ*S*_*max*_. This stems from the fact that for realistic systems −Δ*S* can never reach the value of −Δ*S*_*max*_ as it would require for one of the values in the entropy difference to be equal to zero. This, in turn, is not realistic as in order for the system to be maximally disordered the temperature would have to approach infinity.

At this point, one can note that all of the above results describing the variation in the magnetic entropy −Δ*S* upon changing the temperature vary from each other depending on the type of the transition which the system is subjected to. In addition to that, in the case of each of the transitions, there is a peak associated with the maximum value of −Δ*S* for a given process that originates from a difference between the entropy of two different thermodynamic states assumed by the system before and after a specific transition. In the case of this work, such transitions correspond to the change in parameters *α*, *B* and *R*_*g*_ that affect the interaction between magnetic nanoparticles. The first of these parameters, i.e. angle *α* which may assume any value at the range between 0° to 180°, affects the distance between nanoparticles, i.e. *d*. This means that upon altering the value of *α* the separation distance between nanoparticles is also being changed. More specifically, upon approaching the value of *α* = 90° (either by increasing *α* at the interval [0°, 90°] or decreasing it at the interval [90°, 180°]), the value of *d* is increasing which stems from the fact that *d* assumes the maximum value for *α* = 90°. Conversely, the larger the difference between the value of *α* and 90°, the smaller the value of *d*. Furthermore, it should be noted that during the transition from the system with maximally separated nanoparticles (*α* = 90°) to the system with densely packed nanoparticles (*α* = 0° or *α* = 180°), the peaks which are shown on graphs of −Δ*S*/*S*_*max*_ plotted against temperature tend to shift towards higher temperatures. A similar observation can also be made for the system subjected to the variation in another parameter, i.e. *B*. More specifically, the transition of the system from the thermodynamic state without magnetic field (*B* = 0T) to the state with a non-zero value of *B* increases the height of peaks observed in the case of graphs showing the dependence of −Δ*S*/*S*_*max*_ on temperature. This, in turn, is caused by the effect of magnetic ordering occurring at the presence of the non-zero external magnetic field. Finally, it should be also noted that the last of the aforementioned parameters, i.e. *R*_*g*_, also affects the way how peaks appearing on graphs of −Δ*S*/*S*_*max*_ versus temperature behave. In this case, the interaction energy between single-domain magnetic nanoparticles is proportional to *M*^2^ which leads to the shift of the peaks on −Δ*S* versus temperature plots. More specifically, the higher the value of *R*_*g*_, the stronger the magnetic ordering between interacting nanoparticles which results in the greater shift of the aforementioned peaks towards high temperatures.

**Figure 3:**
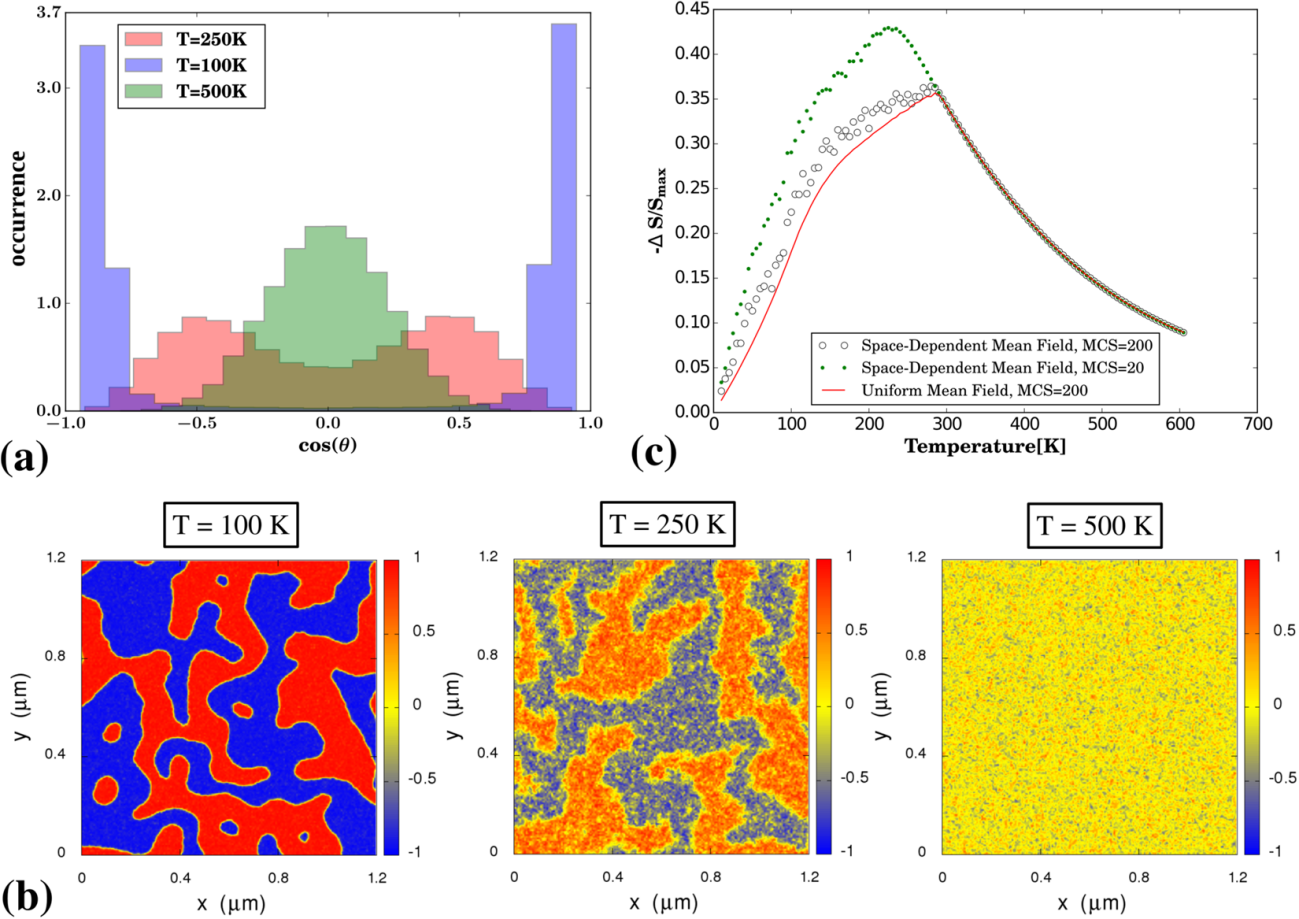
Panels show (a) histograms of the values of cos(*θ*) calculated for the angle *θ* between the space-dependent mean-field magnetic moments and the *z*-axis in a system composed of *L* × *L* magnetic nanoparticles (*L* = 200,*α* = 0°) with *R*_*g*_ = 3 nm after 200 Monte Carlo steps performed at zero magnetic field in the case of three different temperatures, (**b**) graphs corresponding to temperatures specified on panel (a) that show differences between the mfa domains in terms of cos(*θ*) at these temperatures and (**c**) the graph showing the dependence of −Δ*S*/*S*_*max*_ vs. temperature upon applying the external magnetic field of 0.1 T to the system (*L* = 30, *α* = 0°) with the the magnetic domains developed at zero magnetic field after 20 MCS and 200 MCS. In the case of panel (c), the results for the uniform mean-field order parameter (no domains) are added for the sake of a comparison with other results.

In addition to the change in the external magnetic field, size of nanoparticles and the variation in the separation distance between interacting magnetic nanoparticles, another factor that can contribute to the MCE in the considered system is a change in magnetic domains structure. As discussed in the literature^41^, separated magnetic nanoparticles incorporated into a non-magnetic matrix often form a domain-like structure where magnetic nanoparticles are correlated into ferromagnetic areas. As shown in Fig. 3(b), the formation of such domains is also evident in the case of computer simulations conducted in this work that showcase magnetic ordering in the system with 200 × 200 lattice sites. Based on the information obtained from simulations, it is possible to prepare histograms that show a distribution of the angle *θ* (see Fig. 1(a)) between the site-dependent mean-field magnetic moment and the *z*-axis in the case of a zero magnetic field. As shown in Fig. 3(a), the observed distribution is bimodal at temperatures below the critical temperature and gaussian above it. It is also worth to emphasise the fact that such network of competing areas of ferromagnetically ordered magnetic nanoparticles undergoes fast magnetic ordering even at the presence of a weak magnetic field. Furthermore, the effect that the magnetic field has on the extent of MCE for the considered nanoparticle-based material is shown in Fig. 3(c). Based on provided results, it is clear that MCE assumes larger values for weaker magnetic ordering. More specifically, the value of −Δ*S* calculated after 20 Monte Carlo steps (MCS) is much larger than after 200 MCS. For comparison, results generated after 200 MCS in the case of a uniform mfa are also provided. At this point, it should also be mentioned that some preliminary studies of the magnetic domain evolution in magneto-mechanical metamaterials subjected to a deformation were already conducted in^60^. The main finding of that work was related to the fact that magnetic domains in systems with a non-magnetic matrix deformed at a fast rate grow to a smaller extent than in the case of systems deformed slowly which is in agreement with results in presented in this work (see Fig. 3). It also implies the importance of the dynamic behaviour of magneto-mechanical structures and the potential of such systems to be used in the design of new refrigeration devices.

All of this is very important as in this work, it is shown that the considered system has a potential to be used in order to design materials capable of exhibiting a strong MCE without a need of a strong magnetic field even at room temperature. More specifically, it was shown that a dense assembly of appropriately arranged magnetic nanoparticles can manifest a strong magnetocaloric effect upon being subjected to a relatively weak change in the external magnetic field. Furthermore, it was discussed that a process of the mechanical deformation corresponding to the change in the distance between interacting nanoparticles can also induce the MCE which effect is possible even without the presence of the external magnetic field. However the additional implementation of the external magnetic field can further enhance the magnitude of the induced MCE. In addition to that, it was shown the the selection of nanoparticles having an appropriate size plays an important role in the process of the optimisation of the extent of the change in the entropy manifested by the system. All of these results indicate that the use of deformable dense assemblies of magnetic nanoparticles may potentially lead to the design of efficient magnetic refrigerators/heaters that are significantly more environmentally friendly than conventional cooling devices. Furthermore, the use of the concept discussed in this work can also prove to be useful in the case of applications requiring the local change in the temperature.

## Conclusions

The results of this work suggest that thin layers of magnetic metamaterials composed of ultra-small nanoparticles can manifest a strong local MCE at room temperature even for a weak external magnetic field which is not the case for known bulk materials. In addition to that, it was discussed that the extent of the observed change in the magnetic entropy can be controlled depending on a number of different parameters such as a range of the mechanical deformation, extent of a change of the external magnetic field or the variation in the size of magnetic nanoparticles. It was also observed that ferromagnetic-like domains, that form in the considered system composed of densely-packed nanoparticles, significantly increase the MCE. Finally, it was discussed that the concept of deformable magneto-mechanical metamaterials is promising from the point of view of future applications such as magnetic refrigeration.

## Methods

### Mean-field metropolis monte carlo algorithm

*L*_*s*_ copies of a magnetic system with the one-body Hamiltonian, described by means of Eq. (1) are defined on a square *L* × *L* lattice, where each copy is composed of *N* magnetic nanoparticles (*N* = *L*^2^) with a magnetic moment ${\overrightarrow{m}}_{i}$ (*i* = 1, …, *N*). Each magnetic moment can interact only with the mean fields ${\overrightarrow{H}}_{j}={K}_{ij}\langle {\overrightarrow{m}}_{j}\rangle$ at the neighbouring lattice sites and these fields are the same for each copy. Before commencing the Metropolis scheme, initially, all magnetic moments ${\overleftarrow{m}}_{i}$ (*m*_*x*_ = sin*θ*cos*ϕ*, *m*_*y*_ = sin*θ*sin*ϕ*, *m*_*z*_ = cos*θ*) within the system are randomly oriented, where *θ* ∈ [0, *π*] and *ϕ* ∈ [0, 2*π*]. The main objective of this method is to determine $\langle {\overrightarrow{m}}_{i}\rangle$ (*i* = 1, …, *N*). To do that, the following procedure is conducted *L*_*s*_*N* times for each Monte Carlo step:

- one magnetic nanoparticle should be chosen at random from the set of *L*_*s*_ copies,
- an attempt should be made to rotate its magnetic moment from $\overrightarrow{m}=({m}_{x},{m}_{y},{m}_{z})$ to ${\overrightarrow{m}}^{\text{'}}=({m^{\prime} }_{x},{m^{\prime} }_{y},{m^{\prime} }_{z})$ with the probability min(1, exp(−*β*Δ*E*)), where Δ*E* = *E*(*m*_*x*_^'^, *m*_*y*_^'^, *m*_*z*_^'^) − *E*(*m*_*x*_, *m*_*y*_, *m*_*z*_) denotes the energy change due to such rotation and *β* = 1/*k*_*B*_*T*,
- if the rotation described in point (ii) actually occurs, then the value of $\langle {\overrightarrow{m}}_{i}\rangle$ in Eq. (2) in the main text should be updated.

### Monte carlo integration algorithm

The integrals in Eqs. (3) and (4) are calculated with the help of the Monte Carlo integration scheme. It is a numerical integration method which uses random numbers to calculate approximate value of the integral under consideration. The standard deviation in this approximation is equal to $\sigma  \sim 1/\sqrt{n}$ for a given number *n* of random numbers. In our case, the random numbers are represented by a sequence of *n* random values of *θ* and *ϕ*. Furthermore, the algorithm used in this work in Eq. (4) in the main text to calculate entropy *S*_*i*_ at the lattice site *i* is the following:

- one should generate a sequence of *n* random values of *θ* and *ϕ*: {(θ_1_, *ϕ*_1_), (θ_2_, *ϕ*_2_), …, (θ_*n*_, *ϕ*_*n*_)},
- the probability sequence {*p*_1_, *p*_2_, …, *p*_*n*_} should be calculated with
  6 $${p}_{k}={{\rm{e}}}^{-\beta {H}_{{\rm{m}}{fa}}^{(i)}({\theta }_{k},{\phi }_{k})}/\frac{2{\pi }^{2}}{n}\mathop{\sum }\limits_{l=1}^{n}\,\sin ({\theta }_{l}){{\rm{e}}}^{-\beta {H}_{{\rm{m}}{fa}}^{(i)}({\theta }_{l},{\phi }_{l})},$$
  where *k* = 1, 2, …, *n* and sin(*θ*_*l*_) is the Jacobian of the transformation from Cartesian to spherical coordinates (see Eq. (3)),
- entropy at the lattice site *i* should be determined as follows:

7 $${S}_{i}/{k}_{B}=-\,\frac{2{\pi }^{2}}{n}\mathop{\sum }\limits_{k=1}^{n}\,\sin ({\theta }_{k}){p}_{k}\,\log \,{p}_{k},$$

where sin(*θ*_*k*_) is the Jacobian of the transformation from Cartesian to spherical coordinates (see Eq. (4))

It should be noted that the larger value of *n* the more accurate the value of *S*_*i*_.

## Data Availability

The datasets generated during and/or analysed during the current study are available from the corresponding author on reasonable request.
